# Sex Differences in Dietary Patterns of Adults and Their Associations with the Double Burden of Malnutrition: A Population-Based National Survey in the Philippines

**DOI:** 10.3390/nu14173495

**Published:** 2022-08-25

**Authors:** Aileen Rodil de Juras, Wan-Chen Hsu, Yu-Yao Cheng, Li-Jung Elizabeth Ku, Tsung Yu, Cheau-Jane Peng, Susan C. Hu

**Affiliations:** 1 Department of Public Health, College of Medicine, National Cheng Kung University, Tainan City 701, Taiwan; 2 Institute of Human Nutrition and Food, College of Human Ecology, University of the Philippines Los Baños, Los Baños 4030, Philippines; 3 Department of Health and Nutrition, Chia Nan University of Pharmacy and Science, Tainan City 717, Taiwan; 4 Department of Senior Welfare and Services, Southern Taiwan University of Science and Technology, Tainan City 701, Taiwan

**Keywords:** dietary patterns, double burden of malnutrition, adults, Philippines, low–middle income country

## Abstract

A dietary pattern transition is a risk factor for the double burden of malnutrition (DBM), but related information is limited. This study aimed to identify sex differences in dietary patterns of adults in a low–middle income country and to examine their association with DBM. A total of 8957 adults (4465 men and 4492 non-pregnant and non-lactating women) who participated in the 2013 Philippine National Nutrition Survey were included in the analysis. Logistic regression models were formulated to investigate the relationship between dietary patterns and DBM. The factor analysis derived seven dietary patterns for males and six patterns for females. Results showed that approximately 30% of Filipino adults suffered from DBM. The rice pattern was associated with lower odds of DBM for males only. The meat and sugar pattern in males and the protein-rich foods, cereal, and sugar pattern in females decreased DBM likelihood. An inverse relationship was observed for the vegetables and corn patterns, wherein females had an increased risk for DBM. Our findings suggest that rice-based and meat-containing patterns could play protective roles in DBM development among adults in the Philippines. Understanding sex-specific dietary patterns can be utilized to guide public health nutrition interventions in the prevention of malnutrition in all its forms.

## 1. Introduction

Dietary patterns are shifting considerably in low- and middle-income countries, as exemplified by the displacement of staple-food-based diets with increased meat, fat, salt, and added sugar intakes. Consequently, the transition in food patterns is a key driver for the double burden of malnutrition (DBM), defined as the co-existence of undernutrition with overnutrition and diet-related non-communicable diseases across the life course [1,2]. Several studies have been conducted using the dietary pattern approach in order to understand the complex etiology of DBM among adults [3,4,5,6,7,8,9]. Evidence suggests that less diverse diets and a traditional dietary pattern were risk factors for individual-level DBM [7,8,9].

The Philippines is continuously facing DBM. In particular, Filipino adults suffer from malnutrition in all its forms [10,11,12]. Transformations in food consumption have also been evident in the country [13]. What is known to date on the nexus of the dietary pattern–double burden of malnutrition is largely on a national scale. Hence, this study aimed to identify the distinct dietary patterns of male and female community-dwelling adults in a low–middle income setting and to examine the relation of dietary patterns to DBM using the Philippines as an example.

## 2. Materials and Methods

### 2.1. Data Source and Subjects

We analyzed the data from the 8th Philippine National Nutrition Survey (PNNS), a cross-sectional study that is accessible through http://enutrition.fnri.dost.gov.ph/site/home.php (accessed on 3 September 2020) [14]. Briefly, the survey was carried out from 2013 to 2014 by the Department of Science and Technology–Food and Nutrition Research Institute to determine the nutrition and health status of Filipinos. The PNNS has a stratified multistage sampling design representative at the national, regional, and provincial levels. The objectives, design, and procedures of PNNS have been detailed elsewhere [15,16].

**Figure 1:**
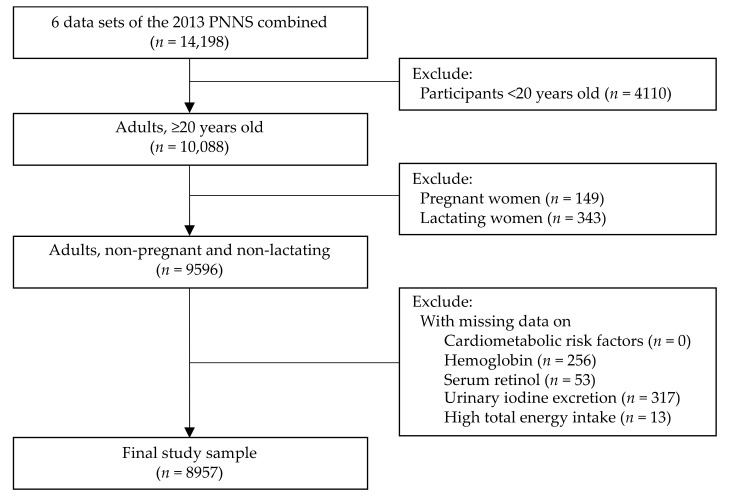
Selection of study participants. (Cardiometabolic risk factors were defined as an individual with any of the following factors: (1) overweight/obesity or abdominal obesity, (2) hypertension, (3) hyperglycemia, or (4) dyslipidemia [low high-density lipoprotein (HDL) cholesterol or hypertriacylglycerolemia]. There were no study participants with missing values on cardiometabolic risk factors).

The study participants were restricted to male and female adults (≥20 years old) with complete subject identification in the six survey components (i.e., dietary, anthropometry, biochemical, clinical, socioeconomic individual, and socioeconomic household). Pregnant women, lactating mothers, and those with missing data on cardiometabolic risk factors (CMRF), hemoglobin, serum retinol, and urinary iodine excretion (UIE) were excluded. Participants with high energy intake (greater than 5 standard deviations of mean energy intake) were also excluded [17]. No participants had low energy intake or lower than 5 standard deviations of mean energy intake. As a result, a total of 8957 adults were included in the analysis. The flowchart of the selection process for the study samples is illustrated in Figure 1.

### 2.2. Dietary Intake Assessment and Dietary Pattern Analysis

Dietary intake was assessed with 24-h food recall in the 8th PNNS. Registered nutritionist-dietitians administered the food recalls on two non-consecutive days. Common household measurements or food sample weighing was utilized to estimate the amount of food and beverages consumed. Calibrated kitchen utensils, rulers, and a photo compilation of foods were used as aids. Subsequently, the weights were converted to purchased values, and energy intakes were computed utilizing the Philippine Food Composition Table. The food items were then aggregated into food groups [18].

Dietary patterns were derived separately for males and females through factor analysis (principal axis factoring method with varimax rotation in R software) based on the mean intake of 18 food groups (Supplementary Table S1). To avoid too many zero values in the data and irrelevant results, only the food groups that were consumed by more than 10% of the study population were included in the analysis [19,20,21,22]. The number of factors retained was determined considering the scree plot results, components with eigenvalue >1.0, and factor interpretability (Supplementary Figure S1). A factor loading of ≥0.25 was the cut-off value for identifying food groups that strongly contribute to a particular dietary pattern [22,23,24]. The naming of dietary patterns was decided according to published studies and data interpretation. Additionally, when a food group was loaded in more than one dietary pattern, the group with the higher or positive coefficient was accounted for in the labeling [25]. Factor scores were then calculated and divided into tertile intervals. The bottom tertile (T1) corresponds to low adherence in a dietary pattern, the middle tertile (T2) corresponds to medium adherence, and the upper tertile (T3) corresponds to high adherence. The Kaiser-Meyer-Olkin measure of sampling adequacy and Bartlett’s test of sphericity were done before factor analysis to evaluate data suitability.

### 2.3. Undernutrition, Cardiometabolic Risk Factors, and Double Burden of Malnutrition

A study participant was considered to be experiencing undernutrition if at least one of the following conditions was present: (1) underweight, (2) anemia, (3) vitamin A deficiency, or (4) iodine insufficiency. An underweight categorization was assessed utilizing body mass index (BMI) and calculated as the weight in kilograms divided by the height in meters squared. The weight and height of the participants were obtained by employing mechanical platform beam balance scales (Detecto™) and microtoise (Seca™), respectively. Furthermore, the BMI classification applied was done according to the World Health Organization (WHO) [26]. Biochemical indicators for three micronutrient deficiencies, i.e., anemia, vitamin A deficiency, and iodine insufficiency, were collected during the survey. Anemia was examined from hemoglobin utilizing a spectrophotometer [27]. Hemoglobin values <13 g/dL for males and <12 g/dL for females indicated anemia [28]. On the other hand, vitamin A deficiency was tested from serum retinol by High-Performance Liquid Chromatography [29] and distinguished as serum retinol <10 µg/dL [30]. Iodine insufficiency was determined from UIE levels through the acid digestion/colorimetric method [31]. The cut-off used was UIE <50 µg/dL [32].

The criteria used for having a CMRF were adopted from Zeba and colleagues [9]. It was defined as having any of the following factors: (1) overweight/obesity or abdominal obesity, (2) hypertension, (3) hyperglycemia, or (4) dyslipidemia [low high-density lipoprotein (HDL) cholesterol or hypertriacylglycerolemia]. Overweight/obesity and abdominal obesity were categorized based on the WHO guidelines [26,33]. Overweight and obesity were evaluated by computing the BMI. For waist circumference, calibrated tape measures were utilized [18]. Hypertension was denoted by a blood pressure measurement of ≥140/≥90 mmHg [34]. Blood pressure readings were performed with a calibrated non-mercurial sphygmomanometer (A&D Um-101™) and stethoscope [18]. Hyperglycemia was characterized as a fasting blood glucose ≥110 mg/dL [35], and dyslipidemia was characterized as having an HDL cholesterol <40 mg/dL for males or <50 mg/dL for females, or triglyceride ≥150 mg/dL [36,37]. Plasma blood glucose was analyzed for hyperglycemia, while serum blood lipids were assessed for dyslipidemia via the enzymatic colorimetric method [18]. We described the total double burden of malnutrition (total DBM) at the individual level as the concomitance of various forms of undernutrition (underweight, anemia, and vitamin A deficiency or iodine insufficiency) and at least one CMRF [12].

### 2.4. Other Co-Variates

The other co-variates in this study were the sociodemographic and lifestyle characteristics obtained through one-on-one interviews. Sociodemographic information encompasses sex (male or female), age (20–39, 40–59, and ≥60 years), educational attainment (elementary and lower, high school, college and higher), marital status (single, married or with partner, and others or widowed/separated/annulled/divorced), employment status (employed or unemployed), and wealth quintile (poorest, poor, middle, rich, richest). Household size was created from the socioeconomic datasets and categorized as 1–3, 4–6, and ≥7. The lifestyle factors of smoking (current smoker or not), alcohol consumption (current drinker or not), and physical activity (low or high) were likewise controlled in the analysis and classified utilizing WHO standards [38,39].

### 2.5. Statistical Analysis

All data analyses were conducted in R software version 4.0.3 (R Foundation for Statistical Computing, Vienna, Austria). The percentages of sociodemographic characteristics, lifestyle factors, and total DBM were generated according to sex and compared using the Chi-square test. A binary logistic regression analysis was employed to evaluate the relationship between the tertiles of dietary pattern scores and total DBM for males and females separately, since there was significant interaction with sex and some outcome variables. The formulated models were adjusted for all co-variates and the energy intake. The reference group for each dietary pattern was the bottom tertile (T1). Multicollinearity was assessed in all models. The multi-level sampling design of the survey was considered in the regression analysis, i.e., sampling weights were employed to generate results representative of the adult population in the Philippines. The significance level was set at *p* < 0.05.

## 3. Results

### 3.1. Participants’ Characteristics

A total of 8957 adults were included in this study with a balance between male and female participants (Table 1). The study sample mostly belonged to the 20–39 years old age group (46.5%), finished high school education (37.9%), were married (66.6%), and were employed (59.5%). There were slightly more females in the older age group (16.6%) and more females who attained college education or higher (32.4%) relative to males. Alternatively, more males were single or unmarried (26.4%) and employed (76.3%). The median household size was four and no sex differences were noted. There were more males in the poorest and poor quintiles than females. In terms of lifestyle factors, 26.9% were current smokers, 51.3% were current alcohol drinkers, and 44.2% had low physical activity. A noticeably greater percentage of males were smokers and alcohol drinkers, whereas more females had low physical activity.

About 36% of the participants were suffering from undernutrition and 84.5% had CMRF with a significant sex difference (Table 2). Iodine insufficiency (23.8%) and low HDL cholesterol (70.1%) had the highest prevalence among the indicators of undernutrition and CMRF, respectively. Correspondingly, the individual-level DBM affected approximately one-third of the adult population (29.5%).

### 3.2. Dietary Patterns

Table 3 and Table 4 present the dietary patterns that were extracted through factor analysis for males and females. Seven dietary patterns explaining 25.5% of the total variance in the consumption of food groups were derived for males. For females, 6 dietary patterns were generated, representing 20.5% of the variance in food intake. The three dietary patterns composed of: (1) the rice pattern (with high positive loading in the rice and rice products food group), (2) the fruits and miscellaneous food pattern (consisting of fruits and other miscellaneous food groups), and (3) the fish pattern (the fish and fish products food group had high factor loading), which were common for both males and females. The results of the factor analysis also demonstrated a number of sex differences. For example, the meat and sugar pattern; the vegetables pattern; the cereal, egg, and oils pattern; and the beverage pattern emerged among males but not females. On the contrary, the dietary patterns labeled as protein-rich food, cereal and sugar, vegetables and corn, and fats and oils were seen only among females.

### 3.3. Association of Dietary Patterns and Double Burden of Malnutrition

The relationship between total DBM and tertiles of dietary pattern scores was examined using a logistic regression analysis. The rice pattern and the meat and sugar pattern were associated with DBM in males (Table 5). Those in the middle tertile (T2) of the rice pattern were less likely to have DBM—after adjusting for sociodemographic characteristics, lifestyle factors, and energy intake, unlike males in the bottom tertile (T1). Similarly, male adults with medium and high adherence (T2 and T3) to the meat and sugar pattern had a lower risk for total DBM. The remaining dietary patterns showed no significant associations with DBM among males.

Regarding females, two dietary patterns were found to be associated with DBM (Table 6). Those in the upper tertile (T3) of the protein-rich foods, cereal, and sugar patterns had a lower likelihood of having DBM in the regression models that controlled for the co-variates. An inverse relationship was noted in the vegetables and corn pattern. Female adults with medium and high adherence (T2 and T3) to the latter pattern had higher odds of developing DBM.

## 4. Discussion

In this study, seven dietary patterns emerged through factor analysis for males and six for females. The rice and fish patterns were also ascertained in previous research [40,41,42,43]. In the same manner, the fruits and miscellaneous food pattern, the vegetables pattern, and the vegetables and corn pattern were consistent with past literature [44,45]. The key food groups in the meat and sugar pattern and cereal, egg, and oils pattern of males and the protein-rich foods, cereal, and sugar pattern of females resembled the dietary patterns pertained as unhealthy [46,47,48], Western [49], and high fat and sugar [50]. The beverage pattern and the fats and oils pattern were described in earlier studies as well [51,52,53,54].

Individual-level DBM affected about three in every ten adults (29.5%) and was higher than the estimates of Zeba and colleagues [9]. Data analysis revealed that dietary patterns had mixed effects on total DBM. Filipino male adults consuming a diet high in rice had a decreased susceptibility for total DBM. The meat and sugar pattern identified among males and the protein-rich foods, cereal, and sugar pattern derived among females were associated with a decreased risk for DBM. Interestingly, the vegetables and corn dietary patterns increased the risk for DBM in females.

Studies on the relationship between rice intake and DBM are scarce and frequently draw on metabolic syndromes or its components as outcomes. In a recent meta-analysis, rice intake was positively correlated with metabolic syndrome [55]. A pooled analysis of three US cohorts observed no associations between white and brown rice consumption and cardiovascular health [56]. Eshak and colleagues [57] likewise explored the relation between white rice and major cardiovascular diseases among men and reported an inverse correlation. The latter study was coherent with our findings. Probable reasons for lower cardiometabolic risk are the varying rice starch compositions [58], processing and cooking methods [58], and complementary dishes eaten with rice [59]. For micronutrient deficiencies, the Philippines has been implementing the fortification of rice with iron since 2000 [60]. Production of healthier rice varieties, such as high-iron and high-zinc types, are also being carried out as part of the biofortification efforts [61]. These recommended public health strategies have been implemented to address micronutrient malnutrition in the country [62]. However, it is important to note that high consumption of rice and rice products alone is not recommended without ensuring diet diversity, and that total carbohydrate intake is within the acceptable macronutrient distribution range.

Male adults adhering to the meat and sugar pattern and female adults favoring the protein-rich foods, cereal, and sugar pattern had a lower likelihood of having DBM. This can be supported on a number of accounts. First, these dietary patterns are comprised of animal proteins, mainly from meat, poultry, milk, and dairy products that have been found to be negatively associated with blood pressure, insulin resistance, and obesity in previous literature [63,64]. Second, animal-based protein foods, specifically meat, are high in heme-iron, zinc, and vitamin B12 [65]. Third, high amounts of sugar and syrups are present in these dietary patterns. Available evidence has illustrated that dietary sugars do not cause obesity and diet-related disease, but rather sugar consumption in excess of energy requirements [66]. Khan and colleagues further substantiated that the kind of sugar, sucrose in particular, was associated with a reduction in cardiovascular disease mortality [67].

It is widely known that vegetable-containing dietary patterns are favorable for lessening the risk of non-communicable diseases due to the dietary fiber, antioxidants, and phytochemicals it contains. These bioactive compounds regulate insulin secretion, lipid profile fluctuations, oxidative stress, and inflammatory and immune status [68,69,70,71,72,73,74,75]. Moreover, some vegetables are rich sources of essential vitamins and minerals, though less bioavailable than animal sources [76]. The positive association between the vegetables and corn pattern and DBM can be substantiated by the low vegetable consumption among Filipino adults, i.e., daily per capita vegetable intake (68.5–68.9 g/day) [77,78], which may have counteracted the hypothesized benefits. Collectively, a balanced dietary pattern with the appropriate combination of food groups should be put forward together with the current nutritional guidelines.

Our findings also had limitations. Firstly, causality and lifetime dietary intake cannot be drawn, given the nature of the study. Secondly, reporting bias and measurement errors are inherent in assessing dietary intakes. Thirdly, factor analysis involves several subjective decisions to be made. Fourthly, the dietary patterns explained the low variability in total food intake (25.5% for males and 20.5% for females). Fifthly, the nutrient intakes of the male and female adults by tertile of the dietary pattern scores were not calculated. Finally, there were unmeasured confounders, thus warranting careful interpretation of the results when generalizing to the general population of adults. Despite these limitations, our study is one of the few nationally-representative epidemiological investigations focusing on the impact of dietary patterns on malnutrition in all its forms among community-dwelling adults.

## 5. Conclusions

In conclusion, this population-based study identified sex-specific dietary patterns that were significantly associated with DBM development among adults in the Philippines. Our findings suggest that rice-based and meat-containing food patterns may potentially exert protection against the risk of developing nutritional deficiencies and cardiometabolic diseases among Filipino adults simultaneously. These unique dietary patterns can be utilized to guide public health nutrition interventions directed toward DBM prevention. Further research is necessary to validate our findings.

## Figures and Tables

**Table 1:** Characteristics of the participants according to sex.

Variables ^1^	Total	Male	Female	*p*-Value
	(*n* = 8957)	(*n* = 4465)	*(n* = 4492)	
Age group				<0.001
20–39 years	46.5	48.8	44.2	
40–59 years	38.3	37.3	39.3	
≥60 years	15.2	13.8	16.6	
Educational attainment				0.005
≤Elementary	31.9	33.9	29.9	
High school	37.9	38.2	37.6	
≥College	30.2	27.8	32.4	
Marital status				<0.001
Single	23.4	26.4	20.4	
Married	66.6	67.9	65.2	
Others	10.1	5.7	14.4	
Employment status				<0.001
Employed	59.5	76.3	43.0	
Unemployed	40.5	23.7	57.0	
Household size				0.840
1–3	33.3	33.3	33.3	
4–6	45.1	44.9	45.3	
≥7	21.6	21.8	21.4	
Wealth quintile				<0.001
Poorest	17.6	19.4	15.8	
Poor	19.3	20.0	18.6	
Middle	20.6	20.7	20.4	
Rich	20.6	19.9	21.2	
Richest	22.0	20.0	24.0	
Current smoker				<0.001
Yes	26.9	46.0	8.1	
No	73.1	54.0	91.9	
Current alcohol drinker				<0.001
Yes	51.3	72.4	30.6	
No	48.7	27.6	69.4	
Physical activity				<0.001
Low	44.2	36.5	51.7	
High	55.8	63.5	48.3	

Values are weighted percentages (%). ^1^ Variables with missing observations: educational attainment (*n* = 44), smoking and drinking status (*n* = 521), physical activity classification (*n* = 614).

**Table 2:** Distributions of undernutrition, cardiometabolic risk factors, and double burden of malnutrition.

Variables	Total	Male	Female	*p*-Value
	(*n* = 8957)	(*n* = 4465)	(*n* = 4492)	
Undernutrition				
Underweight	11.2	10.4	12.0	<0.001
Anemia	6.5	5.4	7.5	0.014
Vitamin A deficiency	0.1	0.0	0.1	0.430
Iodine insufficiency	23.8	21.0	26.5	0.001
Cardiometabolic risk factors				
Overweight/Obesity	29.4	25.9	32.9	0.001
Abdominal obesity	13.4	3.4	23.2	<0.001
Hypertension	22.5	24.3	20.6	0.008
Hyperglycemia	10.2	11.1	9.3	0.047
Low HDL cholesterol	70.1	61.4	78.8	<0.001
Hypertriacylglycerolemia	39.5	46.5	32.6	<0.001
≥1 Undernutrition ^1^	35.5	32.1	38.9	0.001
≥1 Cardiometabolic risk factor ^2,3^	84.5	81.9	87.1	0.002
Total DBM ^4^	29.5	25.6	33.3	<0.001

Values are weighted percentages (%). ^1^ Having any of the following conditions: (1) underweight, (2) anemia, (3) vitamin A deficiency, (4) iodine insufficiency. ^2^ Having any of the following factors: (1) overweight/obesity or abdominal obesity, (2) hypertension, (3) hyperglycemia, (4) dyslipidemia (low HDL cholesterol or hypertriacylglycerolemia). ^3^ Cardiometabolic risk factor with missing observations: both body mass index and abdominal obesity (*n* = 220), hypertension (*n* = 36), hyperglycemia (*n* = 352), both low high-density lipoprotein (HDL) cholesterol and hypertriacylglycerolemia (*n* = 110). ^4^ Total DBM, total double burden of malnutrition or the co-existence of underweight or anemia or vitamin A deficiency or iodine insufficiency and at least one cardiometabolic risk factor.

**Table 3:** Factor loadings for the seven dietary patterns identified among males.

Food Groups	Dietary Patterns ^1^						
	Rice	Meat and Sugar	Fruits and Miscellaneous Food	Fish	Vegetables	Cereal, Egg, and Oils	Beverage
Rice and rice products	**0.964**	0.071	0.007	0.077	0.076	−0.002	−0.038
Corn and corn products	−0.411	−0.022	−0.029	0.043	0.236	−0.118	−0.007
Other cereal products	−0.026	0.252	0.032	−0.056	−0.066	**0.381**	−0.031
Starchy roots and tubers	−0.067	0.017	0.015	0.008	0.120	−0.008	−0.008
Sugar and syrups	0.027	**0.460**	0.005	−0.018	−0.025	0.172	−0.007
Dried beans, nuts, and seeds	0.034	0.016	−0.014	−0.074	0.021	0.065	0.021
Green leafy and yellow vegetables	−0.035	−0.091	0.024	−0.012	**0.547**	−0.079	−0.012
Other vegetables	0.099	−0.079	0.024	−0.134	**0.282**	0.074	−0.001
Fruits	0.005	0.028	**0.548**	0.043	0.061	0.052	−0.015
Fish and fish products	0.113	−0.001	0.001	**0.860**	−0.072	−0.012	0.012
Meat and meat products	0.057	**0.351**	0.009	−0.159	−0.101	0.117	0.262
Poultry	0.058	0.174	−0.011	−0.115	−0.036	0.141	0.079
Eggs	0.092	0.002	−0.002	−0.079	−0.065	**0.307**	0.016
Milk and milk products	−0.026	0.083	0.097	−0.023	0.021	0.228	0.037
Fats and oils	0.019	0.041	0.013	0.050	0.022	**0.377**	0.018
Beverages	−0.025	0.103	−0.009	0.008	−0.008	0.041	**0.531**
Condiments and spices	−0.012	0.178	0.026	0.111	−0.016	−0.004	0.063
Other miscellaneous	0.016	0.005	**0.639**	−0.013	0.012	0.051	0.005
Proportion variance, %	6.4	2.8	4.0	4.6	2.7	3.0	2.0
Cumulative variance, %	6.4	9.2	13.2	17.8	20.5	23.5	25.5

Bold values represent food groups kept in their related dietary pattern. ^1^ Dietary patterns are labeled based on the factor loadings with the value of 0.25 or greater.

**Table 4:** Factor loadings for the six dietary patterns identified among females.

Food Groups	Dietary Patterns ^1^					
	Rice	Protein-Rich Foods, Cereal, and Sugar	Fruits and Miscellaneous Food	Fish	Vegetables and Corn	Fats and Oils
Rice and rice products	**0.889**	−0.308	0.019	0.131	0.029	0.114
Corn and corn products	−0.299	−0.067	−0.043	0.028	**0.327**	−0.055
Other cereal products	−0.040	**0.456**	−0.028	−0.001	−0.078	0.122
Starchy roots and tubers	−0.031	0.028	0.065	0.002	0.149	−0.012
Sugar and syrups	0.057	**0.374**	0.056	−0.007	0.040	0.125
Dried beans, nuts, and seeds	0.032	0.037	−0.042	−0.057	0.070	0.205
Green leafy and yellow vegetables	0.018	−0.150	−0.010	0.010	**0.405**	−0.032
Other vegetables	0.074	−0.030	0.053	−0.118	**0.331**	0.112
Fruits	0.000	0.112	**0.536**	0.012	0.080	−0.011
Fish and fish products	0.097	−0.093	0.028	**0.669**	−0.091	−0.070
Meat and meat products	0.051	**0.385**	0.011	−0.156	−0.057	0.110
Poultry	0.047	**0.303**	0.081	−0.050	−0.042	0.069
Eggs	0.045	0.046	0.058	−0.055	−0.041	0.189
Milk and milk products	−0.040	**0.345**	0.107	0.036	0.007	0.063
Fats and oils	−0.018	0.135	0.035	0.090	−0.024	**0.362**
Beverages	−0.028	0.206	0.033	−0.074	−0.031	0.022
Condiments and spices	−0.010	**0.344**	−0.045	0.100	−0.017	−0.087
Other miscellaneous	0.030	0.036	**0.457**	0.002	0.051	0.057
Proportion variance, %	5.1	5.2	3.0	3.0	2.5	1.7
Cumulative variance, %	5.1	10.3	13.3	16.3	18.8	20.5

Bold values represent food groups kept in their related dietary pattern. ^1^ Dietary patterns are labeled based on the factor loadings with the value of 0.25 or greater.

**Table 5:** Logistic regression models for double burden of malnutrition across tertiles of dietary pattern scores among males ^1^.

Dietary Patterns	Total DBM ^2^ (*n* = 1250)
	OR (95% CI)
Rice pattern (ref. = tertile 1)	
Tertile 2	**0.82 (0.68, 0.99)**
Tertile 3	1.06 (0.80, 1.41)
Meat and sugar pattern (ref. = tertile 1)	
Tertile 2	**0.78 (0.64, 0.96)**
Tertile 3	**0.75 (0.58, 0.97)**
Fruits and miscellaneous food pattern (ref. = tertile 1)	
Tertile 2	0.99 (0.82, 1.19)
Tertile 3	0.96 (0.79, 1.16)
Fish pattern (ref. = tertile 1)	
Tertile 2	1.02 (0.86, 1.22)
Tertile 3	1.00 (0.82, 1.23)
Vegetables pattern (ref. = tertile 1)	
Tertile 2	0.97 (0.80, 1.18)
Tertile 3	1.12 (0.91, 1.38)
Cereal, egg, and oils pattern (ref. = tertile 1)	
Tertile 2	0.89 (0.73, 1.09)
Tertile 3	0.93 (0.75, 1.16)
Beverage pattern (ref. = tertile 1)	
Tertile 2	1.05 (0.87, 1.25)
Tertile 3	0.93 (0.75, 1.14)

^1^ Values in bold are significantly different at a level of *p* < 0.05. Models were adjusted for sociodemographic characteristics, lifestyle factors, and energy intake. ^2^ Total DBM, total double burden of malnutrition or the co-existence of underweight or anemia or vitamin A deficiency or iodine insufficiency and at least one cardiometabolic risk factor.

**Table 6:** Logistic regression models for double burden of malnutrition across tertiles of dietary pattern scores among females ^1^.

Dietary Patterns	Total DBM ^2^ (*n* = 1654)
	OR (95% CI)
Rice pattern (ref. = tertile 1)	
Tertile 2	1.16 (0.97, 1.39)
Tertile 3	1.02 (0.80, 1.30)
Protein-rich foods, cereal, and sugar pattern (ref. = tertile 1)	
Tertile 2	0.94 (0.77, 1.15)
Tertile 3	**0.78 (0.61, 0.99)**
Fruits and miscellaneous food pattern (ref. = tertile 1)	
Tertile 2	0.99 (0.82, 1.19)
Tertile 3	0.99 (0.82, 1.18)
Fish pattern (ref. = tertile 1)	
Tertile 2	0.95 (0.79, 1.13)
Tertile 3	0.86 (0.71, 1.04)
Vegetables and corn pattern (ref. = tertile 1)	
Tertile 2	**1.28 (1.07, 1.53)**
Tertile 3	**1.36 (1.12, 1.64)**
Fats and oils pattern (ref. = tertile 1)	
Tertile 2	0.84 (0.69, 1.02)
Tertile 3	0.83 (0.66, 1.03)

^1^ Values in bold are significantly different at a level of *p* < 0.05. Models were adjusted for sociodemographic characteristics, lifestyle factors, and energy intake. ^2^ Total DBM, total double burden of malnutrition or the co-existence of underweight or anemia or vitamin A deficiency or iodine insufficiency and at least one cardiometabolic risk factor.

## Data Availability

Publicly available data sets were analyzed in this study. This data can be found here: http://enutrition.fnri.dost.gov.ph/site/home.php (accessed on 3 September 2020).

## Supplementary Materials

The following supporting information can be downloaded at: https://www.mdpi.com/article/10.3390/nu14173495/s1, Table S1: Food groups used in the dietary pattern analysis; Figure S1: Scree plots showing the eigenvalues of components extracted using factor analysis by sex.
