# Supplementary material for: Sex Differences in Dietary Patterns of Adults and Their Associations with the Double Burden of Malnutrition: A Population-Based National Survey in the Philippines
Source: Nutrients. 2022 Aug 25;14(17):3495. doi: 10.3390/nu14173495 (PMC9460200; doi:10.3390/nu14173495)
Supplement: Supplementary file 1 [file nutrients-14-03495-s001.zip › nutrients-1858726-supplementary.pdf]

Supplementary Files

Table S1. Food groups used in the dietary pattern analysis [1].

Food groups	Food items included
Rice and rice products	Rice, other rice products such as rice noodles, rice cakes
Corn and corn products	Milled corn, corn on a cob, other corn products such as cornstarch, corn pudding, popcorn, corn chips
Other cereal products	*Pandesal*, bread, cookies/biscuits, cakes/pastries, noodles, flour, and others
Starchy roots and tubers	Sweet potatoes and products, potatoes and products, cassava and products, and other roots and tubers such as yam, taro, and arrowroot
Sugar and syrups	Sugars, jams, candies, honey, sweetened soda, sherbet, ice drop, ice candy, sugary foods such as chocolates, and others
Dried beans, nuts, and seeds	Mungbean and products, soybeans and products, nuts and products, and other dried beans/seeds and products such as almond, peas, sesame seed, green peas, tofu, and others
Green leafy and yellow vegetables	Green leafy vegetables, squash fruit, carrot, and other yellow vegetables
Other vegetables	Eggplant, string beans, bitter gourd, other wild vegetables, and other canned/processed vegetables
Fruits	Mango, citrus fruits, strawberry, guava, banana, watermelon, melon, jackfruit, pineapple, young coconut, and others
Fish and fish products	Fresh fish, dried fish, processed fish, crustaceans, and mollusks
Meat and meat products	Fresh meat, organ meat, and processed meat
Poultry	Chicken, other fowls such as duck, goose, pigeon, turkey
Eggs	Hen's egg, duck's egg, other eggs such as quail egg, turkey egg
Milk and milk products	Fresh whole milk, evaporated milk, recombined milk, powdered milk, and condensed milk, cheese, other milk products such as ice cream, yogurt, cultured milk
Fats and oils	Cooking oil, coconut meat, coconut cream, pork drippings and lard, butter, margarine, peanut butter, and others
Beverages	Coffee, tea, alcoholic beverages, chocolate-based beverages, fruit-flavored drink, and others
Condiments and spices	Salt, vinegar, catsup, and other seasonings
Other miscellaneous food	Lemongrass, bay leaves, oregano, turmeric, food coloring, and others

[1] The food groups and food items were pre-identified in the 2013 Philippine National Nutrition Survey.

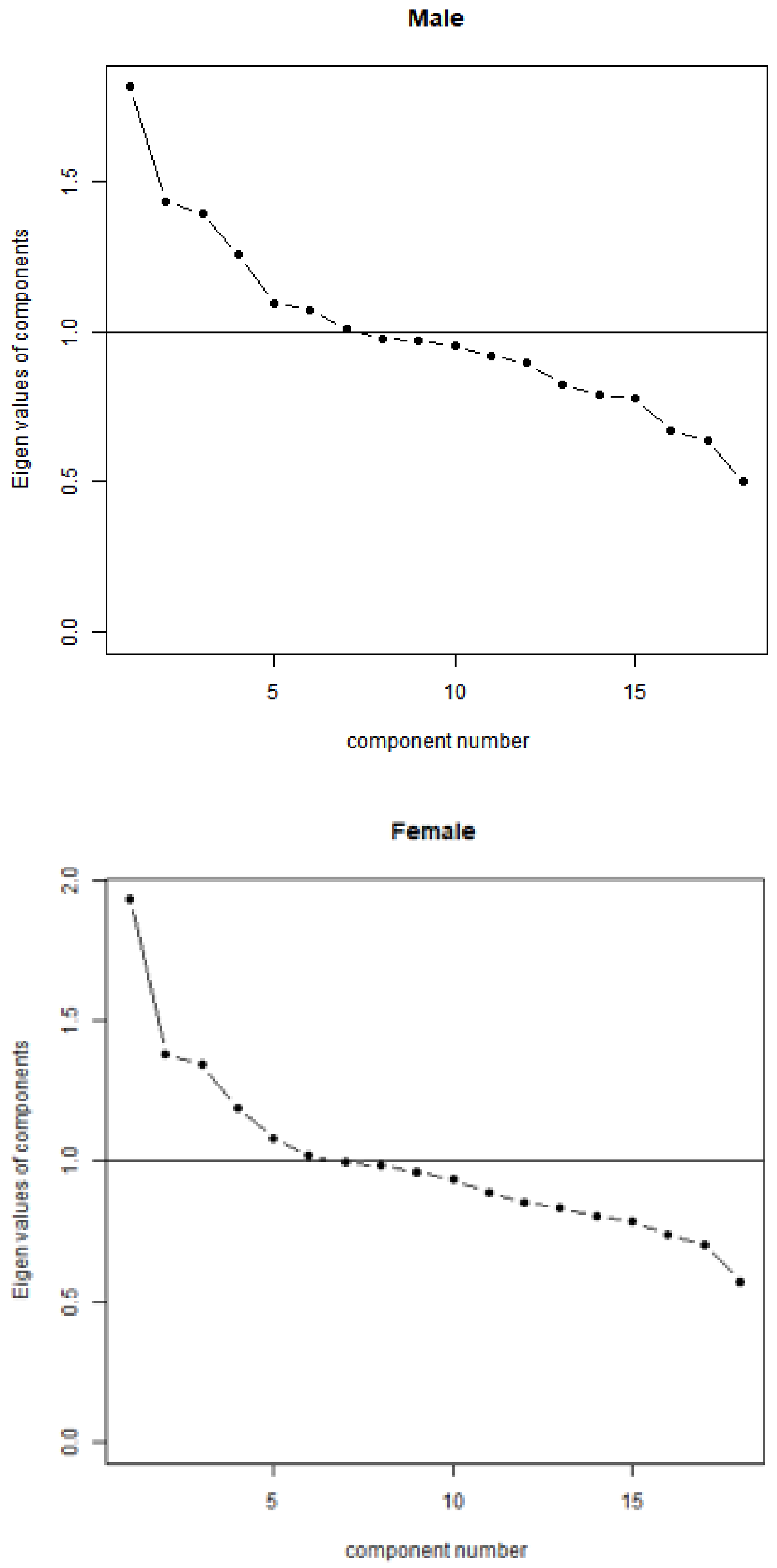

Figure S1. Scree plots showing the eigenvalues of components extracted using factor analysis by sex.
